# PIV investigation of the flow fields in subject-specific vertebro-basilar (VA-BA) junction

**DOI:** 10.1186/s12938-019-0711-9

**Published:** 2019-09-06

**Authors:** Guangyu Zhu, Yuan Wei, Qi Yuan, Jian Yang, Joon Hock Yeo

**Affiliations:** 1 School of Energy and Power Engineering, Xi’an Jiaotong University, No. 28 Xian Ning West Road, Xi’an, 710049 China; 2 Department of Radiology and Medical Imaging, The First Affiliated Hospital of Xi’an Jiaotong University, 277 Yanta Weest Road, Xi’an, 710061 China; 3 School of Mechanical and Aerospace Engineering, Nanyang Technological University, 50 Nanyang Avenue, Singapore, 639798 Singapore

**Keywords:** PIV, In-vitro, Hemodynamics, VA-BA

## Abstract

**Background:**

As the only arterial structure of which two main arteries merged into one, the vertebro-basilar (VA-BA) system is one of the favorite sites of cerebral atherosclerotic plaques. The aim of this study was to investigate the detailed hemodynamics characteristics in the VA-BA system.

**Methods:**

A scale-up subject-specific flow phantom of VA-BA system was fabricated based on the computed tomography angiography (CTA) scanning images of a healthy adult. Flow fields in eight axial planes and six radial planes were measured and analyzed by using particle image velocimetry (PIV) under steady flow conditions of ${Re}=300$, ${Re}=500$. A water–glycerin mixture was used as the working fluid.

**Results:**

The flow in the current model exhibited highly three-dimensional characteristics. The confluence of VAs flow formed bimodal velocity distribution near the confluence apex. Due to the asymmetrical structural configuration, the bimodal velocity profile skewed towards left, and sharper peaks were observed under higher Reynolds condition. Secondary flow characterized by two vortices formed in the radial planes where 10 mm downstream the confluence apex and persists along the BA under both Reynolds numbers. The strength of secondary flow under ${Re}=500$ is around 8% higher than that under ${Re}=300$, and decayed nonlinearly along the flow direction. In addition, a low momentum recirculation region induced by boundary layer separation was observed near the confluence apex. The wall shear stress (WSS) in the recirculation area was found to be lower than 0.4 Pa. This region coincides well with the preferential site of vascular lesions in the VA-BA system.

**Conclusions:**

This preliminary study verified that the subject-specific in-vitro experiment is capable of reflecting the detailed flow features in the VA-BA system. The findings from this study may help to expand the understanding of the hemodynamics in the VA-BA system, and further clarifying the mechanism that underlying the localization of vascular lesions.

## Introduction

The vertebro-basilar (VA-BA) system is the only arterial structure in human that two large arteries merge into one, in which the VAs are arising from the subclavian arteries and join into BA. It provides a critical cerebral blood supply path that feeding the posterior circulation of the circle of Willis under normal conditions, and responsible for supplying compensational blood flow to anterior circulation when anatomical or pathological variations occurred [1–3].

Clinical observations have shown that the VA-BA region is a preferential site of vascular lesions. The prevalence of plaques in this region is around 50% in general population [4–8]. Moreover, approximately 25% of ischemic strokes were related to the lesions in VA-BA [9]. Compare with other causes of ischemic stroke, strokes caused by lesions in VA-BA would result in a much higher in-hospital mortality (8% vs. 20%) and worse functional outcomes [10].

Hemodynamics characteristics have long been associated with the initiation and the progression of the vascular diseases [4, 11–13]. Fry et al. [14] firstly suggested that the wall shear stress (WSS) excess 40 Pa could result in acute damages in the endothelial layer of vessels. In contrast, Caro et al. indicated that early lesions prefer to develop in low WSS regions [15]. This observation is supported by in-vivo and in-vitro studies that concerned hemodynamic at the carotid bifurcations [16, 17], coronary arteries [18–21], and descending thoracic aorta [22]. The results from the above studies have shown that the intimal thickening of vessels is strongly correlated with regions of low WSS. Further support of the atherogenic role of low WSS came from animal models [23, 24], computational fluid dynamics (CFD) simulations [10, 25], and studies at molecular and cellular scales [26, 27]. Based on the above evidence, low WSS theory is currently considered to be more convictive than high WSS hypotheses. In addition to low WSS, oscillatory shear stress [16, 17, 22, 28] and spatial wall shear stress gradient (SWSSG) [29] are also implicated as critical adverse factors in the disease process.

Most of the studies, however, are concerning the blood flow in bifurcations. Only limited attention has been paid to the flow characteristics in the arterial confluence. Despite the geometry of bifurcations is similar to junctions, the hemodynamic patterns in the arterial confluences are totally different from bifurcations due to the reversed flow direction, especially near the apex region.

One of the first studies concerning the flow in the VA-BA system was conducted by McDonald et al. [30]. In their in-vivo animal experiment, flows in the BA were visualized by injecting ink into the vessels. The study showed that the streams from symmetrical VAs would not mix in the BA. Limited by the method itself, however, no quantitative data were provided. Thereafter, multiple research methods were applied in the investigation of hemodynamic characteristics in VA-BA. Numerically, simulations were performed by applying two-dimensional models [31–33], three-dimensional symmetric models [31, 34, 35] and patient-specific models [10, 36]. Clinically, the using of MRA made the in-vivo measurement of flow in the confluence area possible [36–38].

In addition to the numerical and in-vivo method, the in-vitro experiment is another powerful research tool to explore the hemodynamics in vascular and to validate the numerical results. Ravensbergen et al. experimentally investigated the impacts of confluence angle [39] and merging flows [34] on flow characteristics in a rectangular cross-section confluence phantom by using laser Doppler anemometry (LDA). The flow velocity profiles in the confluence area were measured, and the results validated the presence of secondary flow in the confluence area that reported in an earlier numerical simulation [35]. Lutz et al. [40] visualized the confluence flow patterns in the VA-BA system by using dye injection, and quantified the mixing effect by introducing the concept of mixing index that based on the measurement of dye concentration. Kobayashi et al. [41] investigated the velocity profiles in VA-BA segments excised from elderly cadavers under different steady flow rates by using high-speed-camera. These studies provided unique and valuable in-vitro perspectives of the flow patterns in the VA-BA region. However, constrained by the measurement tools, certain hemodynamic characteristics in the confluence region, such as shear stress and velocity field, are yet to be fully investigated.

This study aims to investigate the detailed hemodynamics characteristics in the VA-BA system. To achieve the goal, a subject-specific 3D VA-BA flow phantom was fabricated base on CTA scanning images, and the detailed flow features in the VA-BA system were investigated through in-vitro experiments under different Reynolds numbers. Hemodynamics parameters in the confluence area, including velocity fields, shear stress distribution, and secondary flow, were analyzed to provide a better understanding of the role of hemodynamics in the localized vascular lesions.

## Methods

### Image acquisition

**Fig. 1:**
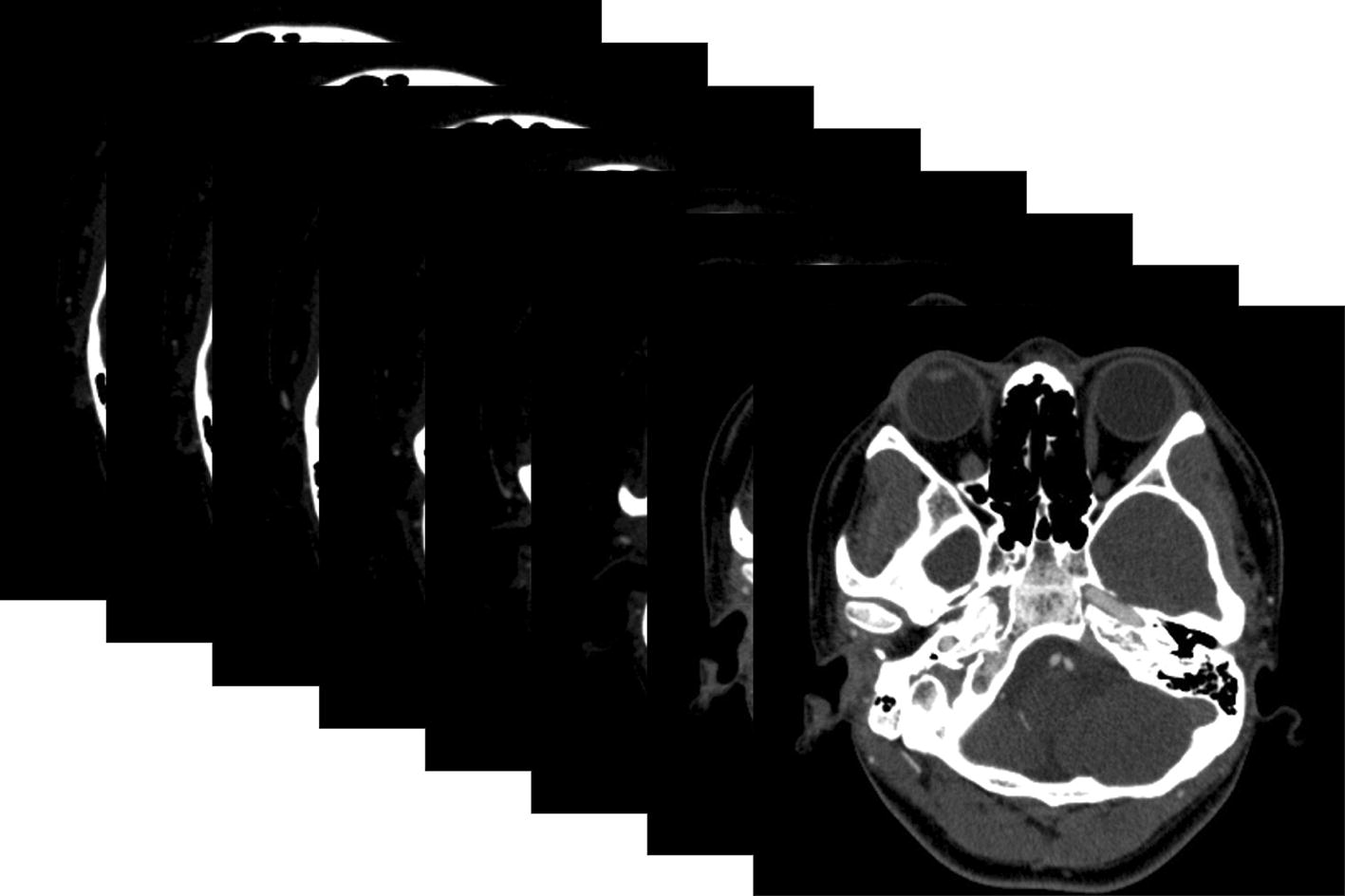
CTA image set of cerebral scanning

The cerebral CTA data of a healthy adult were provided by the First Affiliated Hospital of Xi’an Jiaotong University (Xi’an, Shaanxi, China). The scanning was performed on a 64 detector spiral CT (Aquilion 64, Toshiba Medical Systems, California, USA). The field of view (FOV), number of slices, tube voltage, tube current, scan time, and slice thickness were $265\,\text {mm}\times 265\,\text {mm}$, 967, 120 kV, 350 mA, 500 ms, and 0.5 mm, respectively (Fig. 1).

### Fabrication of the flow phantom

**Fig. 2:**
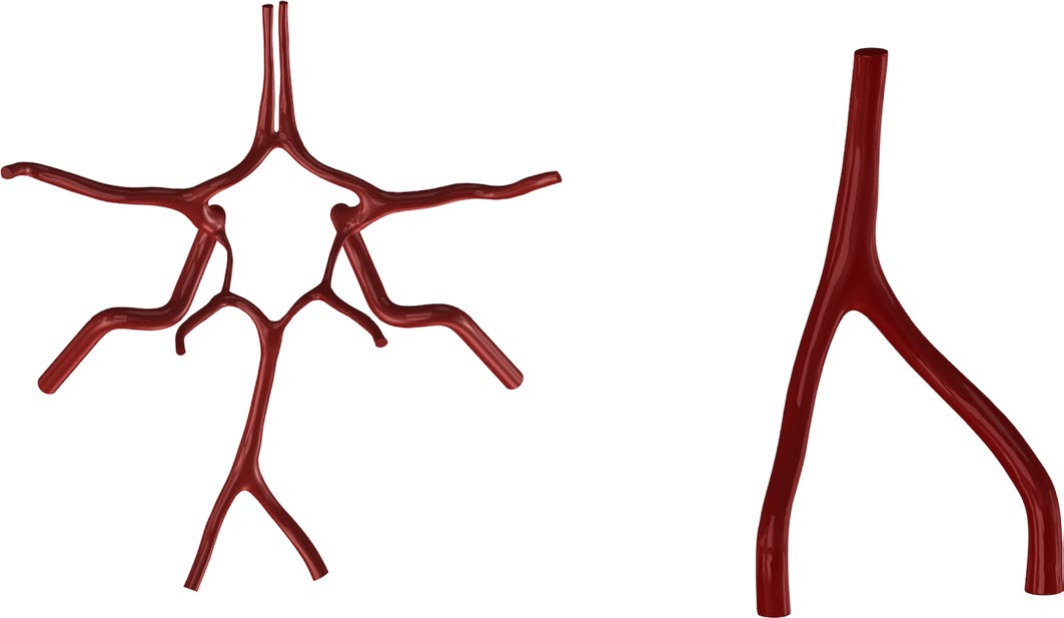
Reconstructed digital model of the VA-BA junction

**Fig. 3:**
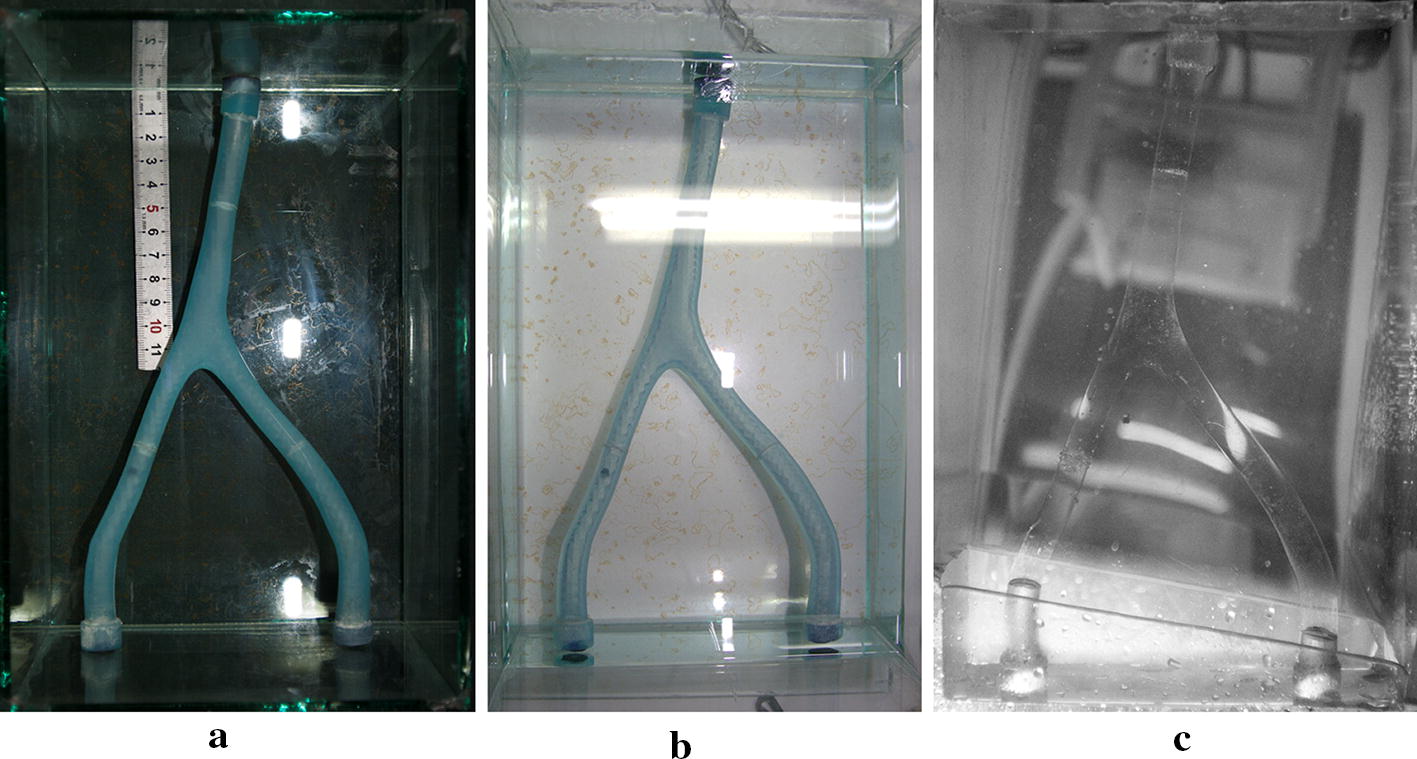
Physical phantom fabrication for experiment

First of all, a 3D digital model of the VA-BA region was reconstructed from the CTA images. The process of reconstruction was introduced in our previous paper in detail [1]. To enable the accurate measurement, the derived VA-BA model was scaled-up to 5 times of original size in Solidworks and exported to STL format (Fig. 2). Then the STL file was sent to the 3D printing system (SCPS350, Xi’an Jiaotong University, China), and a resin phantom of the vascular structure was printed in a resolution of 0.1 mm (Fig. 3a). Finally, the printed vascular model was fixed into a small tank fulfilled with silicone gel (Sylgard 184, Dow Corning Inc., USA) (Fig. 3b). After the curing of the silicone, the resin vascular model was melted by heating, and a transparent phantom with internal flow tunnel was prepared for the in-vitro PIV measurements (Fig. 3c).

### Experimental setup

**Fig. 4:**
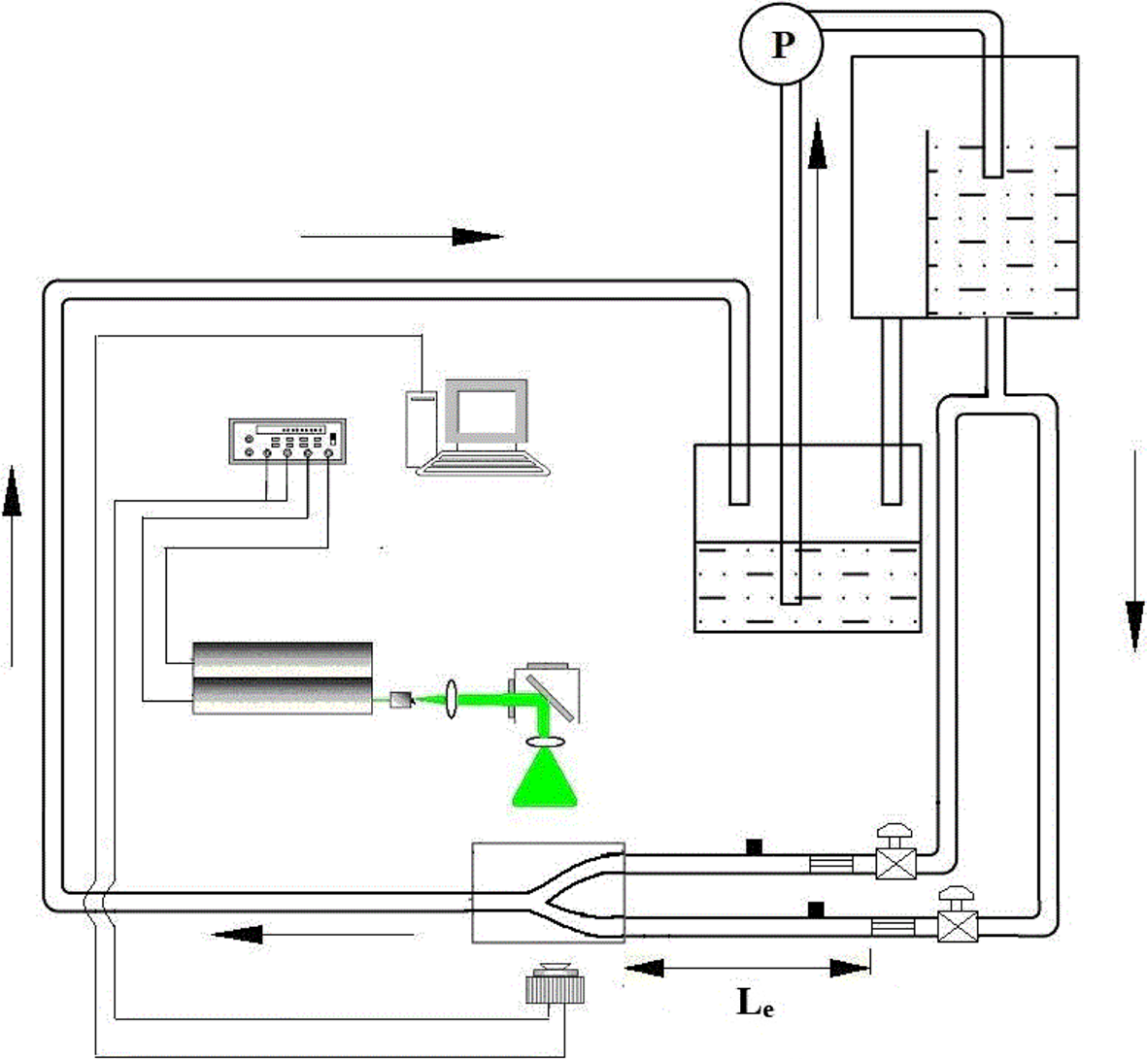
Experimental setup

The experimental setup was composed of a flow loop and a measurement system (Fig. 4).

The steady flow was supplied by an upstream overflow tank that provided constant pressure. According to the empirical equation (Eq. 1) [6], the length of tube that connected the tank was set to 1.6 m and flow-regulating honeycombs were put inside the tubes to ensure the inlet flow fully developed.

1 $$\begin{aligned} L_{\text{e}}=0.06 \times D \times Re \end{aligned}$$

The inlet flow rates of bilateral VAs were controlled by adjusting the valves placed at the upstream of the VA entrances and monitored by the electromagnetic flow meters. At the efferent of BA, an adjustable resistor was connected into the flow loop. The efferent flow of the phantom was collected in a downstream tank and pumped back to the overflow tank. An adjustable thermostat heater was placed in the overflow tank to control the temperature of the working fluid.

### Working fluid and flow parameters

Blood is a multiphase fluid that behaves shear-thinning property. However, the viscosity of blood is relatively constant at shear rates above 100 s^−1^ [7]. Non-Newtonian nature of blood was neglected in the present study because of the shear rates in large arteries as ICA and VA are rarely lower than 100 s^−1^.

**Fig. 5:**
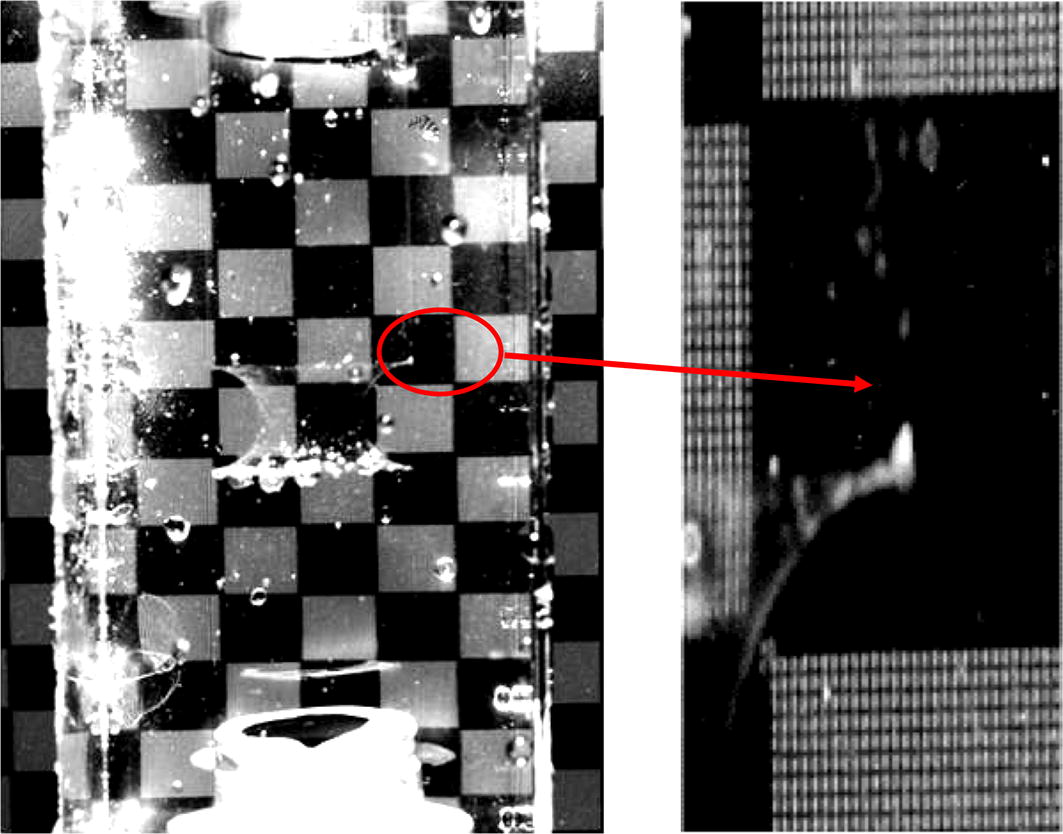
Image distortion after refractive matching

The working fluid that selected for this experiment was the mixture of glycerin and water with a density of 1157 kg/m^3^, a viscosity of 10.6 cP at $37\,^{\circ }\text {C}$ and a refractive index of 1.41. The refractive index of the working fluid matched well with the silicon phantom, and no distortion was observed at the fluid-solid interface (Fig. 5). Hollow glass spheres with an average diameter of $10\,\upmu \text {m}$ were seeded into the working fluid for PIV data acquisition.

Matching the Reynolds number in the experimental condition with the in-vivo arterial flow condition is the key to obtain meaningful results from a scaled flow phantom. As the Reynolds number ranges from 100 to 1000 in medium-size arteries, Reynolds number 300 and 500 were chosen as the experimental conditions. The Reynolds number was defined as follows:

2 $$\begin{aligned} Re=\frac{\rho UD}{\mu } \end{aligned}$$

### Flow visualization

PIV system was used to capture the flow velocity in the phantom. The PIV system consisted of a CCD camera (LaVision Image Pro 4M CCD, $2048\times 2048$ pix^2^), a 200 mJ Nd:Yag laser (Gemini 200, 532 nm), and an optic lens that produces light sheet about 1 mm thick.

To investigate the hemodynamics characteristics in the VA-BA system in detail, the flow fields in eight planes parallel to the flow direction (axial planes) (Fig. 5a) and six planes orthogonal to the mainstream (radial planes) (Fig. 5b) were captured. The axial planes were evenly spaced from top to bottom with an interval of 1 mm. The radial planes were distributed from upstream to downstream with an interval of 5 mm.

The two-frame cross-correlation method was used for image acquisitions. 100 image pairs were recorded in each acquisition to diminish the error caused by random events. The acquired raw images in the IM7 format were post-processed in the DaVis software to obtain the velocity vector files. The interrogation windows size was set to $32\times 32$ pix with 50% overlap. A self-developed MATLAB algorithm was utilized to eliminate the noise outside the flow region.

### Shear stress conversion

Shear stress is defined as follow:

3 $$\begin{aligned} \tau _{xy}=\tau _{yx}=\mu \left( \frac{ \partial v }{\partial x}+\frac{ \partial u }{\partial y}\right) \end{aligned}$$

Thus, shear stress can be got from the velocity vector field. To compare the shear stress in this scaled phantom to that of the original size, a scale factor was applied in this study. The scale factor is derived from Buckingham Pi theorem [10, 42]:

4 $$\begin{aligned} \tau _{v}=\left( \frac{\rho _{b}}{\rho _{f}}\right) \left( \frac{v_{b}}{v_{f}}\right) \tau _{b} \end{aligned}$$

## Results

### Geometrical structure of the VA-BA system

The current VA-BA model has an asymmetrical structural that the sagittal plane of the body did not pass through the center of junction apex. The original diameters of the left VA (LVA), right VA (RVA), and BA were 2.37 mm, 2.75 mm, and 2.83 mm, respectively. The diameter ratio of the bilateral VAs was 1.16 and the confluence angle was $63^\circ$.

### Axial flow patterns

**Fig. 6:**
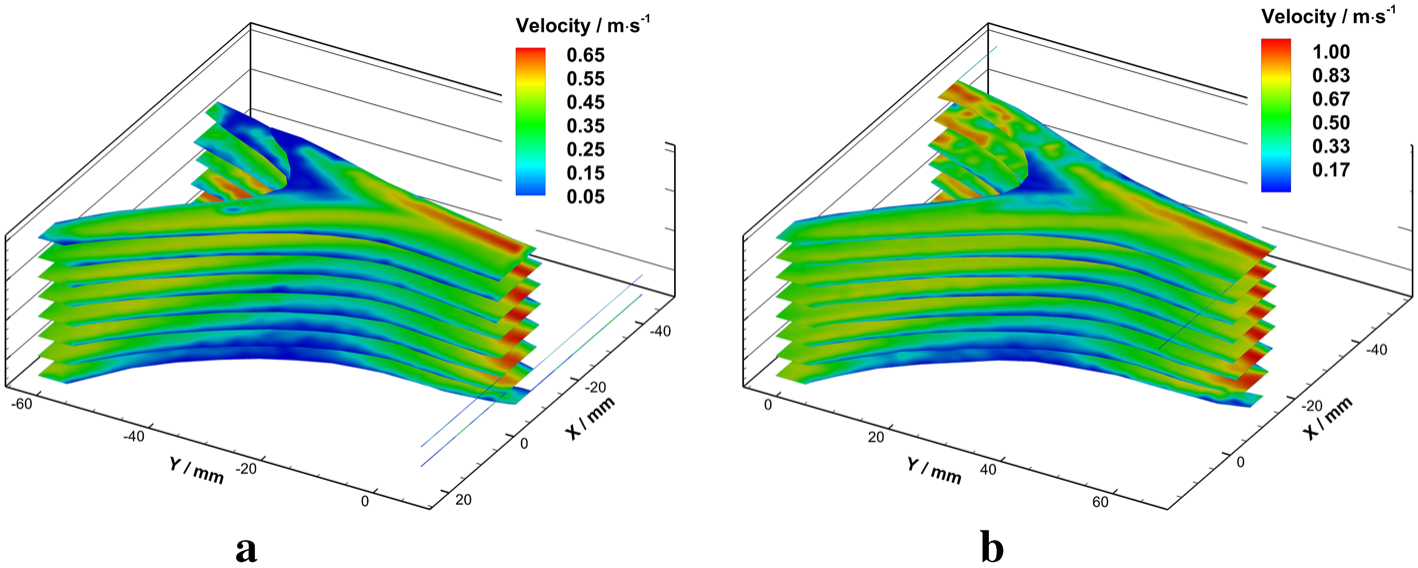
Schematic diagram of velocity fields in axial planes under ${Re} = 300$ (**a**) and ${Re} = 500$ (**b**)

Figure 6 showed the overall axial velocity distributions in the VA-BA region under different Reynolds numbers.

**Fig. 7:**
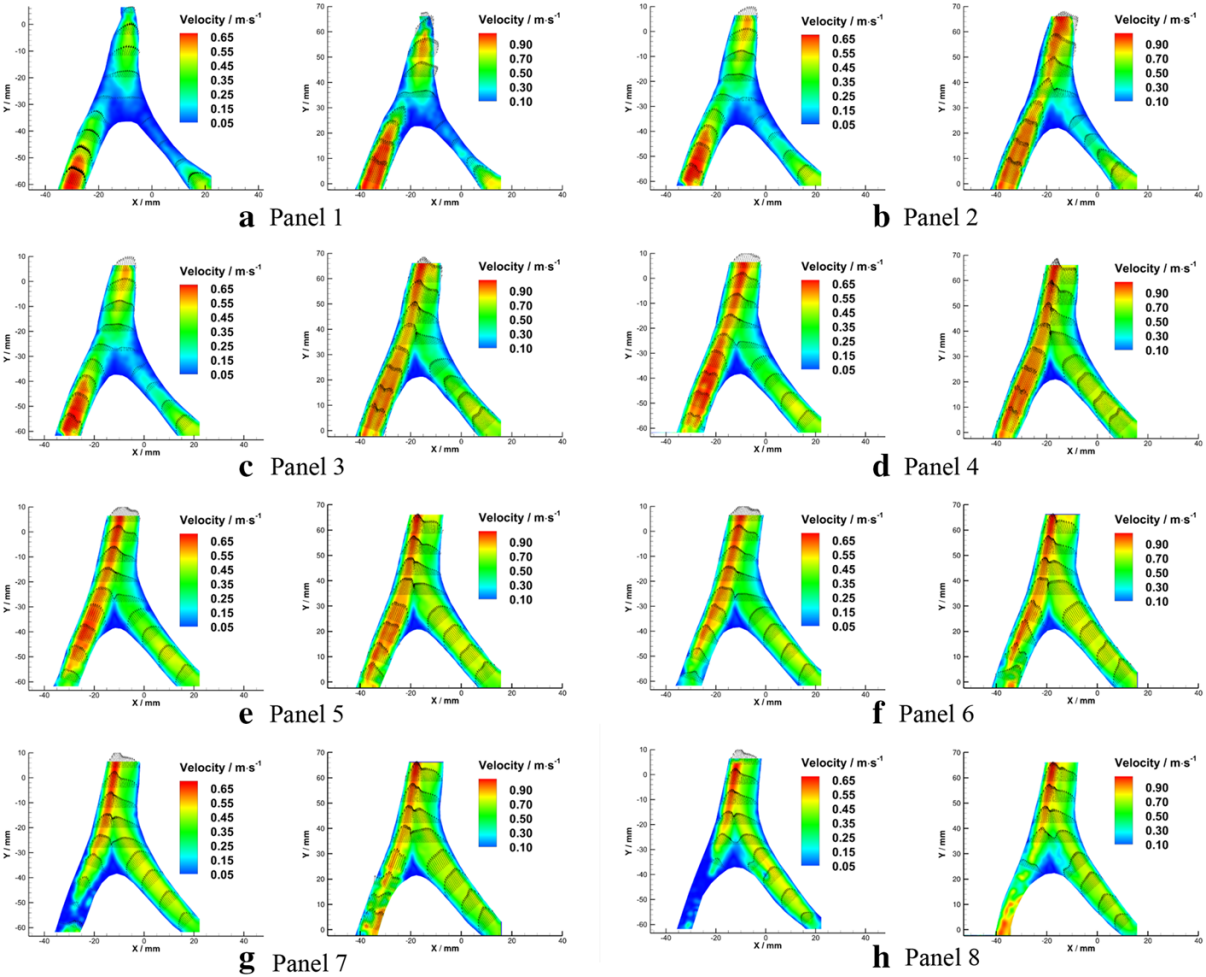
Velocity fields and velocity profiles in axial planes under ${Re}=300$ (left) and ${Re}=500$ (right)

Detailed axial velocity fields (solid contour) and velocity profiles (vector arrows) in each plane were illustrated in Fig. 7.

As the figure showed, the velocity distributions in the bilateral VAs were quasi-parabolic. When the flows confronted, a bimodally distributed velocity profile with a trough at the arterial axis appeared in BA. Under the flow condition of ${Re}=300$, it takes 18 mm in the median plane for the bimodal velocity profile restore to the quasi-parabolic distribution (Fig. 7d, e). In contrast, the bimodal velocity distribution pattern existed all along the BA segment of flow phantom under the flow condition of ${Re}=500$. The peaks of bimodal velocity distribution are more bias to the left side under higher Reynolds number. In addition, a triangle shaped flow stagnation region was found at the confluence apex, where the flow velocity magnitude is below 0.05 m/s. The area of the flow stagnation decreased with the increasing of Reynolds number, which is 74.09 mm^2^ under ${Re}=300$ and 59.69 mm^2^ under ${Re}=500.$

**Fig. 8:**
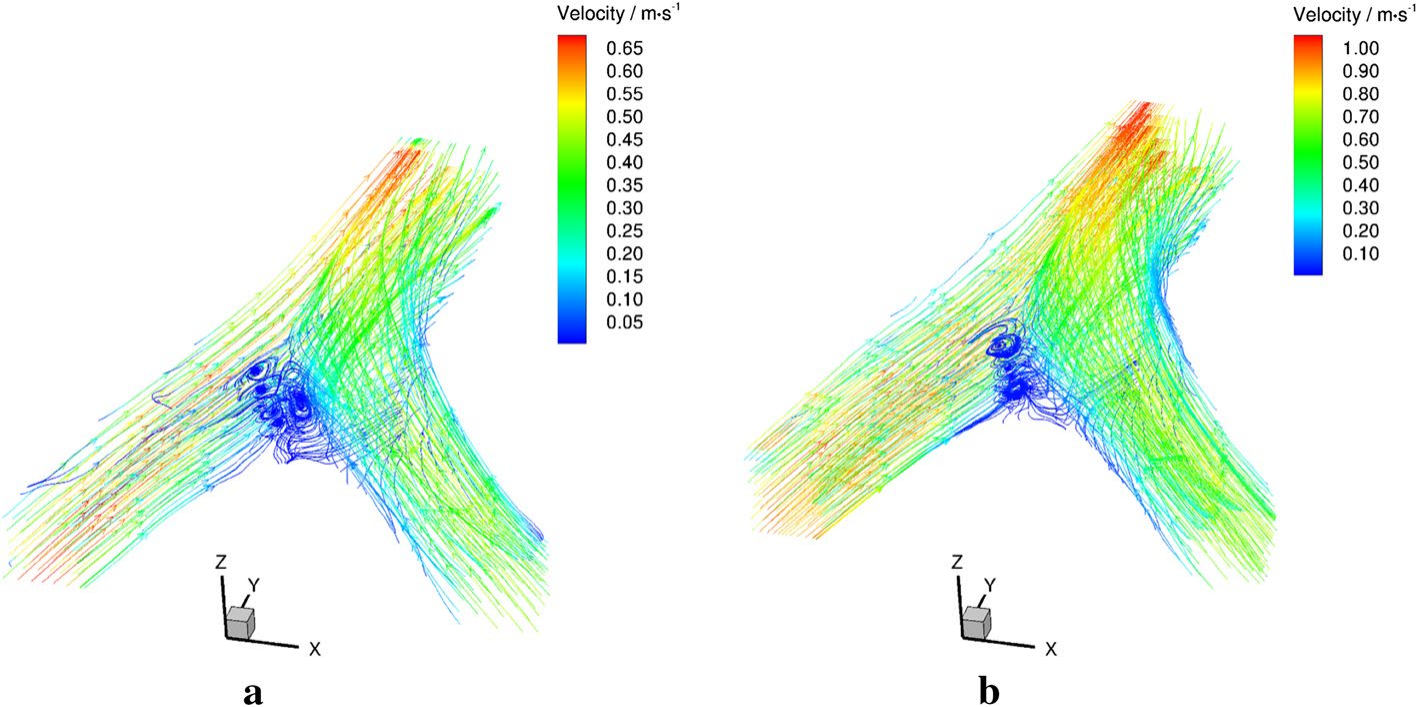
Schematic diagram of streamlines in axial planes under ${Re} = 300$ (**a**) and ${Re} = 500$ (**b**)

Streamlines over the whole VA-BA region under different Reynolds numbers were illustrated in (Fig. 8).

**Fig. 9:**
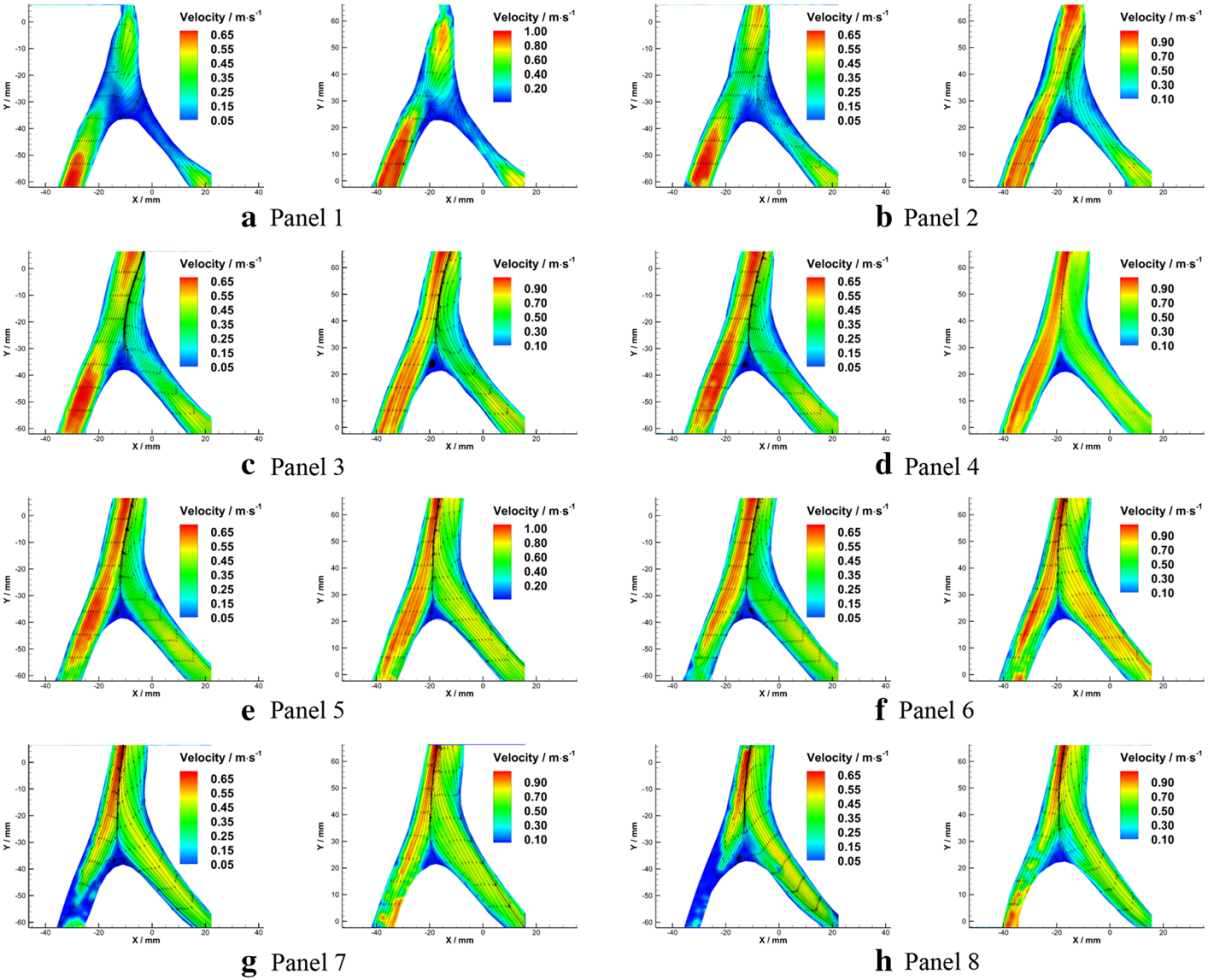
Velocity fields and velocity profiles in axial planes under ${Re}=300$ (left) and ${Re}=500$ (right)

Detailed streamlines in each plane were illustrated in Fig. 9. The overall streamline distributions were similar between different Reynolds numbers. The flows from the bilateral VAs confronted each other at the beginning of the BA where 10 mm downstream the confluence apex. In the upper planes, stream from the left side fully dominated the flow in the BA (Fig. 9a, b). When comes to the lower planes of the flow phantom, the flow from RVA gradually took dominance in the BA (Fig. 9c–h), this phenomenon became stronger as the Reynolds number increases. However, no signs of flow disturbance and mixing were observed in the entire flow region. The two streams from the bilateral VAs remain parallel in BA, and a clear boundary between the bilateral streams could be observed. Moreover, it is interesting to note that stable recirculation zones formed at the bifurcation site while no shedding was observed under experimental conditions.

**Fig. 10:**
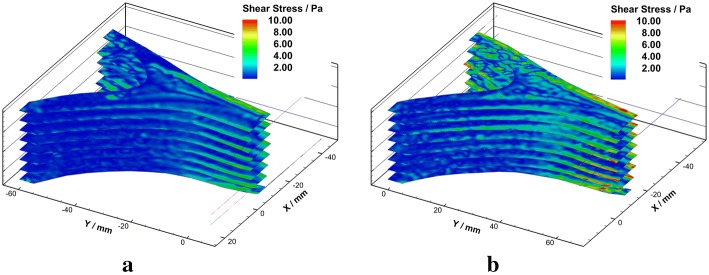
Schematic diagram of shear stress in axial planes under ${Re} = 300$ (**a**) and ${Re} = 500$ (**b**)

**Fig. 11:**
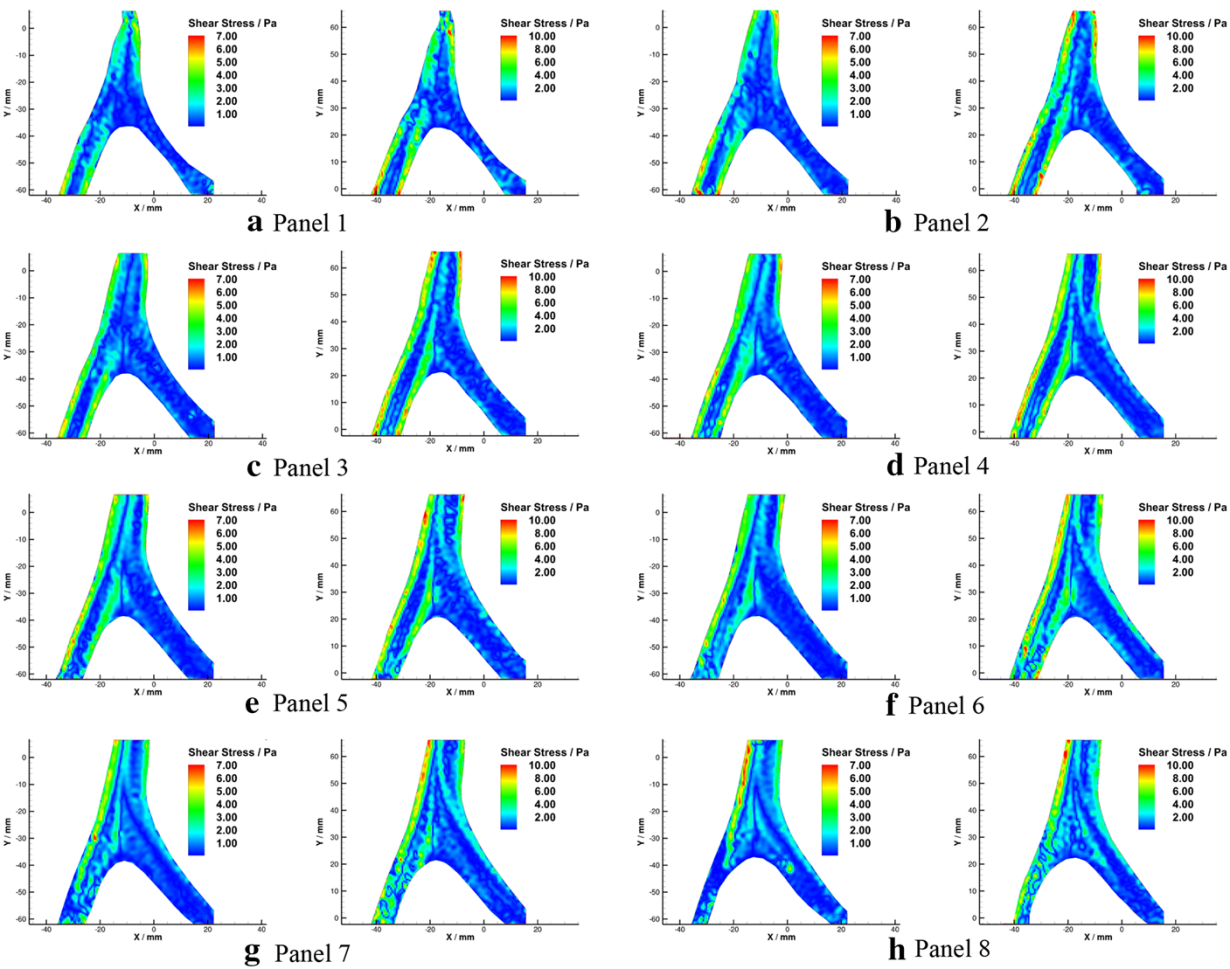
Shear stress in axial planes under ${Re}=300$ (left) and ${Re}=500$ (right)

Shear stress obtained from the experimental data has been converted based on Eq. 4 (Fig. 10). Detailed shear stress distributions in each plane under different Reynolds numbers were plotted in Fig. 11. Low shear stress $(<0.4 \; \text {Pa})$ was observed near the wall of the confluence apex and the left side of the BA. High shear stress was observed along with the confluence interface and the vessel walls of VA and BA.

### Radial flow patterns

**Fig. 12:**
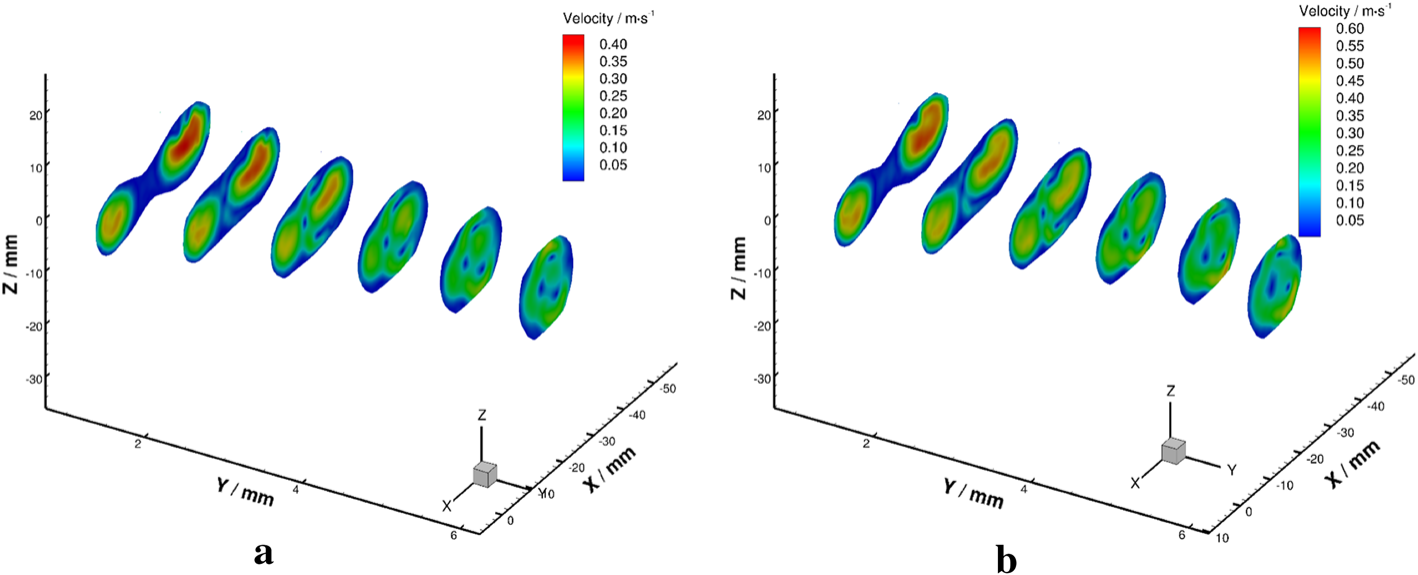
Schematic diagram of velocity fields in radial planes under ${Re} = 300$ (**a**) and ${Re} = 500$ (**b**)

**Fig. 13:**
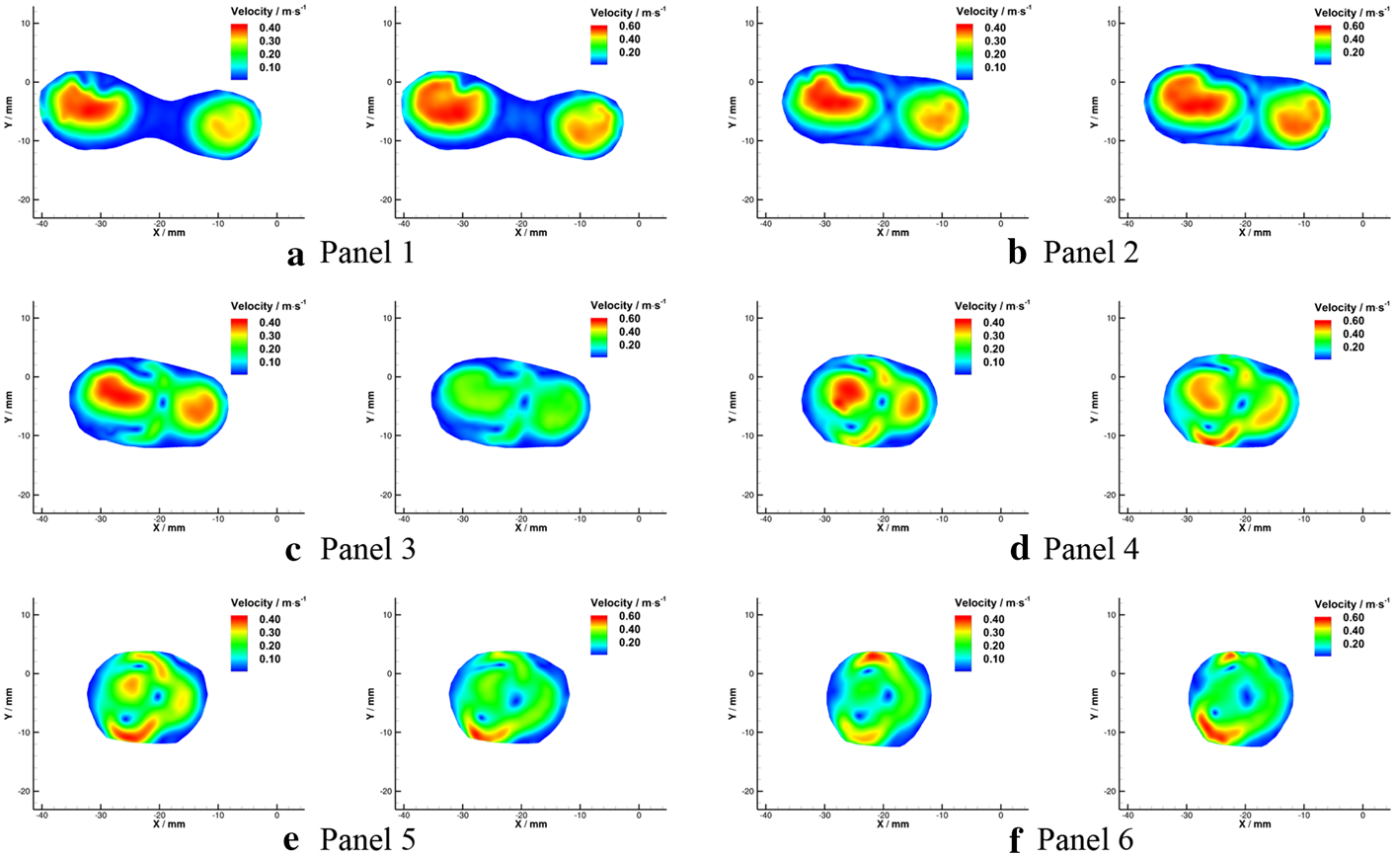
Velocity fields in radial planes under ${Re}=300$ (left) and ${Re}=500$ (right)

Velocity fields in the radial planes were shown in Figs. 12 and 13. Flow from the left VA was wrapped by that from the right side and formed unique three-dimensional flow patterns downstream the confluence apex. An obvious boundary between the flow from the bilateral VAs was observed in the BA (Fig. 13c–f). This phenomenon agrees with the observations in the axial planes.

**Fig. 14:**
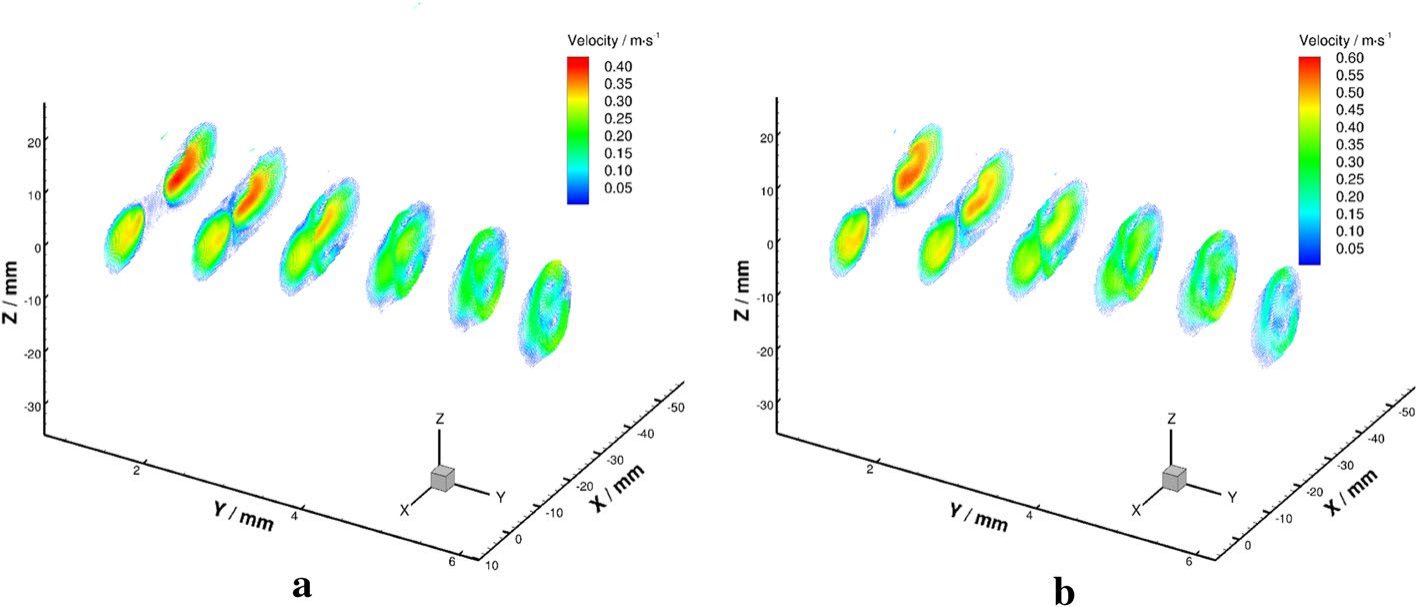
Schematic diagram of velocity vectors in radial planes under ${Re} = 300$ (**a**) and ${Re} = 500$ (**b**)

**Fig. 15:**
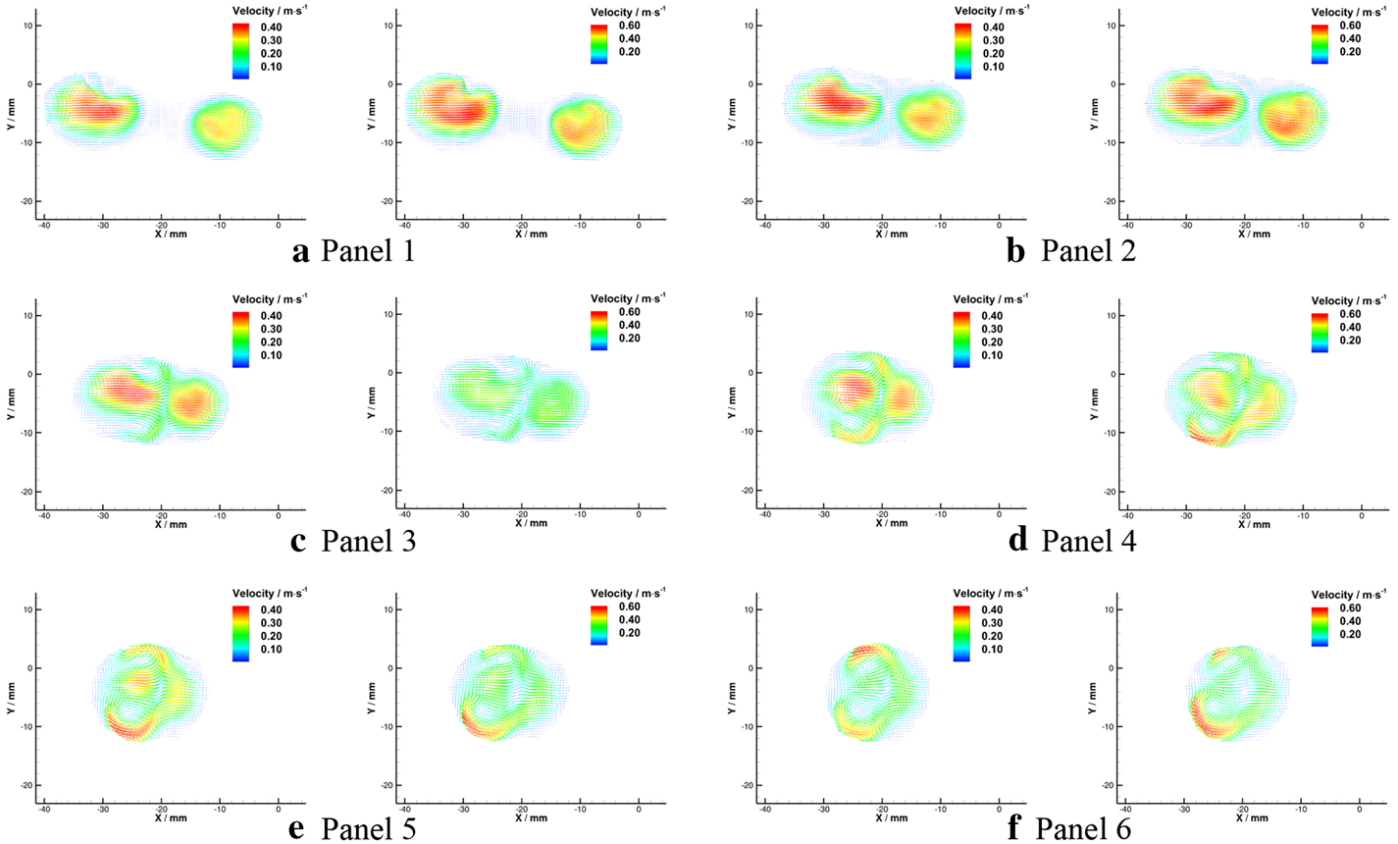
Velocity vectors in radial planes under ${Re} = 300$ (left) and ${Re} = 500$ (right)

**Fig. 16:**
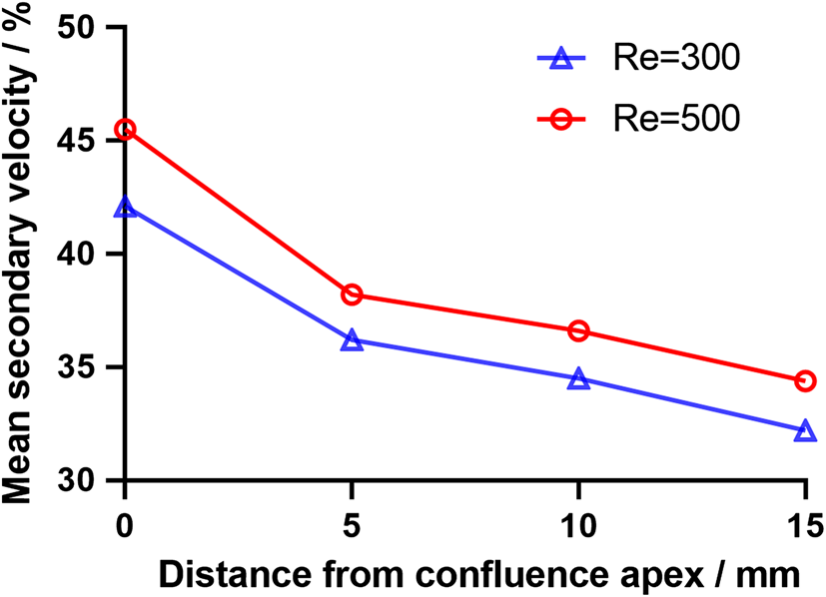
Impacts of Reynolds number and distance from confluence apex on mean secondary velocity

The velocity vectors in radial planes were illustrated in Figs. 14 and 15 further clarified the radial flow characteristic in the VA-BA region. It can be seen that the secondary flows in the BA were characterized by two vortices rotate in opposite directions (Fig. 15c–f). To quantify the strength of the secondary flow, a dimensionless term “mean secondary velocity” that introduced by Ravensbergen et al. [39] was utilized in this study. The mean secondary velocity is defined as the ratio of the cross-sectional mean of the secondary velocities to the cross-sectional mean of the axial velocities at each location. The impacts of Reynolds numbers and distance from the confluence apex on the strength of secondary flow were quantitatively analyzed and showed in Fig. 16, and the specific data were listed in Table 1.

**Table 1:** Mean secondary velocity under different Reynolds numbers

Distance from the confluence apex/mm	Mean secondary velocity/%	
	${Re}=300$	${Re}=500$
10	42.1	45.5
15	36.2	38.2
20	34.5	36.6
25	32.2	34.4

**Fig. 17:**
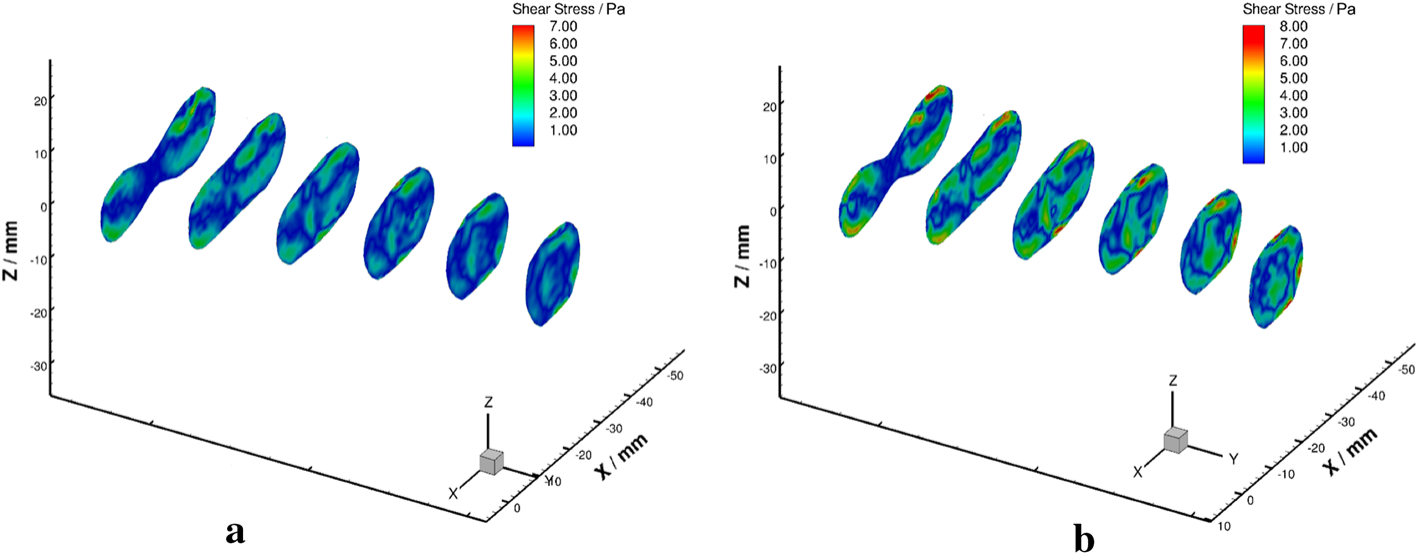
Schematic diagram of shear stress in radial planes under ${Re} = 300$ (**a**) and ${Re} = 500$ (**b**)

**Fig. 18:**
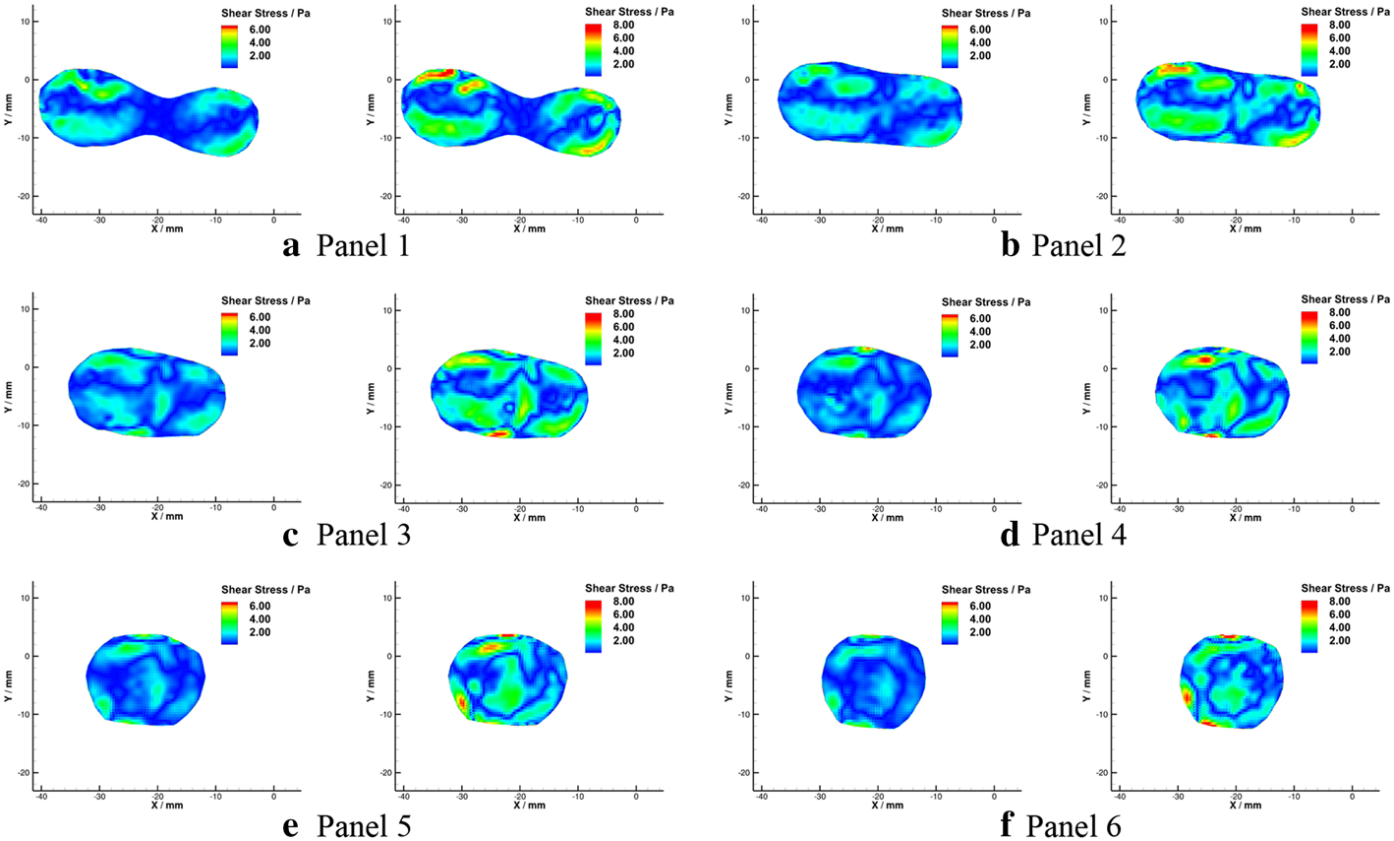
Shear stress in radial planes under ${Re}=300$ (left) and ${Re}=500$ (right)

Shear stress distributions in radial planes were calculated and illustrated in Figs. 17 and  18. It was noticed that a low shear stress zone appeared at the apex site in the radial flow (Fig. 18b) as well, with the value that lower than 0.4 Pa.

## Discussion

In the present study, a subject-specific VA-BA phantom was reconstructed, and *in-vitro* investigations were conducted to elucidate the hemodynamics features of the VA-BA arterial system. Detailed flow patterns and shear stress distributions in the confluence region were studied and compared under different Reynolds numbers.

### Structural characteristics

The geometrical structure of the VA-BA arterial system varies among populations. Previous clinical studies have reported that the confluence angle of the VAs ranges from $10\,^{\circ }$ to $160^{\circ }\,(60^{\circ }{\pm }30^{\circ })$ [10, 39]. Wake-Buck et al. [10] classified the structure of VA-BAs into three types according to the spatial configurations, including Walking type, Tuning fork type, and Lambda type. A recent retrospective clinical study showed that 64% of patients have a Lambda type VA-BA, and the prevalence of Walking type and Tuning fork type configuration is 17% and 19%, respectively [43]. In present study, the confluence angle between the VAs was $63^{\circ }$ and the diameter ratio of RVA to LVA was 1.16, which are within the reported physiological range. The angle between the LVA and BA was $176^{\circ }$ and RVA joints the BA in a pseudo-T-junction, which could be classified as the lambda type VA-BA.

### Flow characteristics

#### Axial flow

As a unique arterial structure, the flow in the VA-BA system also exhibited distinctive characteristics. When confronted in the proximal end of the BA, the streams from the bilateral VAs flowed on their own side without mixing. Such patterns suggested the flow in the BA is laminar. In addition, bimodal velocity profiles appeared immediately after the confluence. Due to the asymmetrical structure, the left peak of the velocity profile is sharper than the right side, which represents the eccentric flow patterns in BA. These findings were supported by several CFD [10], in-vitro [41] and in-vivo [30, 38, 44] studies.

To make the data suitable for quantitative comparison, the normalized velocity that defined as the maximum magnitude in each velocity profile (*U*_max_) divided by the averaged cross-sectional velocity (*U*_mean_) in BA were calculated, and data were listed in Table 2. According to the Poiseuille law, the ratio of *U*_max_ to *U*_max_ should be 2 in the fully developed laminar flow inside a tube, and the length required for the flow development was given in Eq. 1. Because of the limited length, it was impossible for the flow in BA fully developed. Thus, normalized velocity would between 1 and 2, and its magnitude would decrease with increasing of Reynolds number. The results from the current study agree well with the theoretical analysis. Meanwhile, due to the eccentric flow induced by the asymmetrical structure, the normalized velocity under ${Re}=300$ was close to 2 near the confluence apex. The similar phenomenon was reported by Kobayashi et al. [41]. The decreasing of normalized velocity along flow direction revealed that the flow development in BA was affected by the confluence of bilateral streams. Moreover, despite the flows from VAs are parallel along the BA in the median planes, (Fig. 9c–f), helical flow was observed in upper (Fig. 9a, b) and lower planes (Fig. 9g, h) near the distal end of BA segment. This observation further suggested the fluid downstream the confluence flows in a layered pattern along radials direction.

**Table 2:** Normalized velocity under different Reynolds numbers

Distance from the confluence apex/mm	*U*_max_/(m/s)		*U*_mean_/(m/s)		*U*_max_/*U*_mean_	
	${Re}=300$	${Re}=500$	${Re}=300$	${Re}=500$	${Re}=300$	${Re}=500$
10	0.65	0.90	0.33	0.57	1.97	1.58
15	0.61	0.88	0.34	0.58	1.79	1.52
20	0.59	0.87	0.35	0.58	1.69	1.50
25	0.58	0.87	0.36	0.59	1.61	1.47

When focused on the confluence apex region, a zone of low shear stress that coincides with low momentum recirculation as the result of boundary layer separation was observed in this study. The area of this region decreased with the increasing of Reynolds number. The WSS in the VA-BA was derived from the shear stress field that one pixel from to the wall, and low WSS $(<0.4\,\text {Pa})$ region at the apex of VAs junction was predicted in this study. This finding agrees with the results from the idealize and patient-specific models [8, 10, 45]. Several studies have suggested low WSS yield atheroprotective range could trigger an inflammatory-cell-mediated pathway that associated with the growth of atherosclerotic plaques [14, 46–48]. The observation of the localized low WSS provided additional hemodynamic evidence of the preferential of vascular plaques in the confluence apex region. Besides, the low-velocity recirculation zone may accelerate the progression of atherosclerosis by gathering and depositing of blood components.

#### Radial flow

Highly three-dimensional flows in the VA-BA system have been reported in literature [8, 10, 34, 39]. Thus, the investigation of flow patterns in radial direction would be important for a more comprehensive understanding of the hemodynamics characteristics in the VA-BA system.

In previous studies, Ravensbergen’s team investigated the radial flow in a generalized tuning fork type VA-BA system by using CFD simulations [34, 35, 39]. Their results showed that secondary flow with a distinct four-vortex pattern appeared in the BA as the consequence of flow confluence. Furthermore, the same team quantitatively analyzed the impact of flow conditions on the secondary flow strength by using the non-dimensional term mean secondary velocity, which suggested that the secondary flow is stronger under higher Reynolds number and decayed along the flow direction.

The present study is the first in-vitro attempt to investigate the secondary flow in the subject-specific phantom of VA-BA system. Our results showed that the secondary flow was established around 10 mm downstream the confluence apex due to the boundary layer separation, and persists along the BA. The secondary flow strength at the plane 10 mm downstream apex was 42.1% under ${Re}=300$ and 45.5% under ${Re}=500$, and decreased along the flow direction in a non-linear pattern (Fig. 16). Though the directly quantitative comparison of secondary strength between studies is difficult due to the use of different model and flow conditions, its distribution patterns in this study agree well with the results from previous ones [34, 35]. However, we only observed two vortices rotate oppositely in the secondary flow. This phenomenon is mainly due to the skewed flow in the BA, which was induced by the lambda type structural configuration.

Though the average shear stress level in the radial planes is low, relatively high shear stress areas appeared at the interface of two streams and near the artery wall of BA. The secondary flow may bring some blood from a relatively low shear stress area to a high shear stress area repeatedly. Blood components in the high and low shear stress cycle may result in blood damage due to fatigue [49]. Meanwhile, secondary flow in the artery could also play a key role in the erosion and endothelial response at the early stage of atherosclerosis pathology [50].

### Simplifications made in this study

Restricted by the viable experimental condition, it is difficult to fully mimic the physiological flow in the VA-BA system. Thus, several simplifications have been made in the current study.

First of all, the inlet flow was assumed as fully developed at the entrance in the current study. Although this assumption is not physiologically correct, it has been widely accepted in in-vitro experimental studies [51, 52] and provided a basis for comparison between studies.

Secondly, due to the deformation of the vessels is small and blood behaves as Newtonian fluid in arteries of the size of the VA-BA system, the arterial wall elasticity and the non-Newtonian properties of blood were neglected.

In addition, despite the blood flow in the vascular system was pulsatile under physiological conditions, several numerical [16, 53] and in-vitro [41, 54, 55] studies have suggested that steady boundary conditions are able to predict the non-temporal related cerebral flow characteristics at the corresponding point of the pulsatile flow profile. Among which, a comparison between pulsatile results [33] and steady results [31] in two-dimensional VA-BA models showed that the most important flow patterns are the same in both cases. Kobayashi et al. [41] further suggested that the VA-BA flow phenomena occurred in pulsatile flow are essentially the same as those found in steady flow. Thus, the steady flow boundary conditions adopted in this study is capable of revealing the flow characteristics in the region of interesting as no temporal hemodynamics terms were involved.

Moreover, the flow phantom utilized in this study was scaled-up. To overcome the spatial restrictions, the scaling of vascular replicas based on the principle of dynamic similarity, also known as dynamic scaling, has been widely used in the in-vitro experiments [56–59]. The dynamic scaling is a well-established concept that ensures the development of flow in scaled phantom identical to the original object. In the incompressible steady flow within rigid domains, the dynamic similarity could be ensured by matching of the Reynolds number. In this study, a scaled-up VA-BA phantom was deployed due to the difficulties of flow visualization in the original size. As described in the method section of this manuscript, the dynamic scaling has been achieved by matching the Reynolds number between the scaled phantom and *in-vivo* conditions. Thus, the measurements from the scaled phantom are capable of reflecting the flow characteristics in the non-scaled situations.

### Limitations and future works

It is important to emphasize that the current study carries inherent limitations associated with the *in-vitro* modeling techniques. Firstly, the arteries that branch out from BA were removed. To investigate the flow in VA-BA system in a more detailed manner, these branch arteries should be included in future work. Secondly, the elasticity of the arterial wall was neglected. As discussed before, though the rigid wall assumption is acceptable in the current study, it still may lead to a slight overestimation of WSS [60]. In addition, more samples should be included in the future investigations to systemically evaluate the impact of various spatial characteristics [21]. Finally, the three-dimensional flow field should be measured in future work to assess the complex spatial flow in the junction site.

## Conclusions

In this study, in-vitro experiments were conducted to investigate the detailed hemodynamics characteristics in a subject-specific VA-BA flow phantom. Flow characteristics in the axial and radial planes were visualized and analyzed by using PIV. The preliminary results showed that the flow in VA-BA system behaves a highly three-dimensional feature, and the flow patterns were affected by the spatial structures and inflow Reynolds numbers. Further, a low WSS region coincides well with the preferential region of atherosclerotic plaques was found near the confluence apex. The findings from this study could help to expand the understanding of the hemodynamics in the VA-BA system, and further clarifying the mechanism that underlying the localization of vascular lesions.

## Data Availability

Not applicable.

## Abbreviations

BA: basilar artery
CFD: computational fluid dynamics
CoW: circle of Willis
CTA: computed tomography angiography
LDL: low density lipoprotein
NURBS: non-uniform rational B-splines
PIV: particle image velocimetry
VA: vertebral artery
